# Dairy Foods and Dairy Protein Consumption Is Inversely Related to Markers of Adiposity in Obese Men and Women

**DOI:** 10.3390/nu5114665

**Published:** 2013-11-20

**Authors:** Karen J. Murphy, Georgina E. Crichton, Kathryn A. Dyer, Alison M. Coates, Tahna L. Pettman, Catherine Milte, Alicia A. Thorp, Narelle M. Berry, Jonathan D. Buckley, Manny Noakes, Peter R. C. Howe

**Affiliations:** 1 Nutritional Physiology Research Centre, University of South Australia, GPO Box 2471 Adelaide, South Australia 5001, Australia; E-Mails: georgina.crichton@mymail.unisa.edu.au (G.E.C.); kate.dyer@unisa.edu.au (K.A.D.); alison.coates@unisa.edu.au (A.M.C.); tpettman@unimelb.edu.au (T.L.P.); catherine.milte@deakin.edu.au (C.M.); Alicia.Thorp@bakeridi.edu.au (A.A.T.); Narelle.Berry@unisa.edu.au (N.M.B.); jon.buckley@unisa.edu.au (J.D.B.); peter.howe@newcastle.edu.au (P.R.C.H.); 2 Spencer Gulf Rural Health School, 111 Nicolson Ave, Whyalla Norrie, South Australia 5608, Australia; 3 CSIRO Food & Nutritional Science, Kintore Ave, Adelaide, South Australia 5001, Australia; E-Mail: manny.noakes@csiro.au; 4 Clinical Nutrition Research Centre, University of Newcastle, Callaghan, New South Wales 2308, Australia

**Keywords:** dairy products, dairy protein, body composition, abdominal fat, obesity

## Abstract

A number of intervention studies have reported that the prevalence of obesity may be in part inversely related to dairy food consumption while others report no association. We sought to examine relationships between energy, protein and calcium consumption from dairy foods (milk, yoghurt, cheese, dairy spreads, ice-cream) and adiposity including body mass index (BMI), waist (WC) and hip circumference (HC), and direct measures of body composition using dual energy X-ray absorptiometry (% body fat and abdominal fat) in an opportunistic sample of 720 overweight/obese Australian men and women. Mean (SD) age, weight and BMI of the population were 51 ± 10 year, 94 ± 18 kg and 32.4 ± 5.7 kg/m^2^, respectively. Reduced fat milk was the most commonly consumed dairy product (235 ± 200 g/day), followed by whole milk (63 ± 128 g/day) and yoghurt (53 ± 66 g/day). Overall dairy food consumption (g/day) was inversely associated with BMI, % body fat and WC (all *p <* 0.05). Dairy protein and dairy calcium (g/day) were both inversely associated with all adiposity measures (all *p <* 0.05). Yoghurt consumption (g/day) was inversely associated with % body fat, abdominal fat, WC and HC (all *p <* 0.05), while reduced fat milk consumption was inversely associated with BMI, WC, HC and % body fat (all *p <* 0.05). Within a sample of obese adults, consumption of dairy products, dairy protein, and calcium was associated with more favourable body composition.

## 1. Introduction

Dairy products such as milk, yoghurt and cheese are nutritious sources of protein, peptides and other nutrients including calcium, vitamin D and potassium. Unfortunately consumption of dairy products may be discouraged by concern about the risk of obesity and cardiovascular disease (CVD). In Australia milk products and dishes are the major food sources of saturated fat, accounting for ~27% of total intake [1]. Given the link between saturated fat (SFA) and CVD [2,3], this may be a reason which may reflect the relatively low consumption rates of dairy products in Australia [1,4]. Despite the fact that dairy foods have previously been reported to increase risk of CVD, coronary heart disease (CHD) and stroke in prospective cohort studies [2,3], several observational and cross-sectional studies have revealed an inverse association between dairy product consumption and CVD [5] and body composition, weight loss and weight gain [6,7,8,9,10].

Recently Kratz and colleagues [11] conducted a systematic literature review of observational studies investigating associations between dairy fat and cardiometabolic health. Interestingly the authors showed that 11 out of 16 studies reported inverse associations between high fat dairy intake and measures of adiposity. Similarly, a recent systematic review and meta-analysis of randomised controlled trials [12] reported increased dairy product intake was associated with greater reductions in fat mass and WC and a greater gain in lean mass than in controls. In fact, increased dairy product consumption intake resulted in 0.72 kg (95% CI: −1.29, −0.14, *p* = 0.01) greater reduction in fat mass, 2.19  cm (95% CI: −3.42, −0.96, *p*-value < 0.001) further reduction in WC and 0.58 kg (95% CI: 0.18, 0.99, *p <* 0.01) gain in lean mass compared with controls. The authors also stated that increasing dairy product intake without energy restriction did not affect body composition but when dairy product consumption was increased as part of an energy restricted diet designed for weight loss, high dairy food consumption resulted in greater weight loss, reduction in body fat mass, WC and greater increase in lean mass compared with controls. Similarly a prospective investigation of 120,887 men and women in the Nurses Healthy Study I and II and the Health Professionals Follow-up Study showed that yoghurt consumption was inversely associated with 4-year weight change. Additionally age-adjusted linear regression identified that whole fat dairy foods were associated with 4-year weight gain whereas low fat dairy foods were associated with 4-year weight loss. Interestingly in another study [13], higher calcium intake was associated with a lower 5-year increase of the BMI and waist circumference in men but not women. Furthermore, in a 5-year period in men only, a higher consumption of dairy foods was associated with a better metabolic profile. The mechanism by which dairy food consumption may improve body composition is not entirely clear however, it has been postulated the benefit may be in part due to calcium which is thought to reduce lipogenesis and increase lipolysis [14]. Other studies have reported a satiating effect of dairy protein consumption [15,16], while other research suggests conjugated linoleic acid, naturally produced in dairy foods, improves weight through increased fat utilization [17], increased satiety and caloric intake [18,19], however at this time the evidence is mixed in support of these hypotheses [20,21,22]. The purpose of this retrospective study was to explore relationships between dairy product consumption and macro/micronutrients from dairy food (namely protein and calcium) and markers of adiposity within an opportunistic population of overweight or obese adults in Australia. No other published studies have explored relationships between intakes of specific dairy foods and direct measures of body composition using dual energy X-ray absorptiometry (DEXA) within an overweight/obese population. Based on current literature, we hypothesise that dairy foods will be inversely associated with markers of adiposity.

## 2. Materials and Methods

### 2.1. Participants

This study was a cross-sectional analysis of overweight/obese adults. Baseline measurements of body composition of volunteers (*n =* 762) who were recruited in regional and metropolitan South Australia for 11 separate dietary intervention trials between 2004 and 2007 at the Nutritional Physiology Research Centre and CSIRO Human Nutrition were compiled into one database. Information about background information on volunteers, volunteer characteristic, inclusion criteria, data collection methods has been published elsewhere [23,24,25,26,27,28,29,30,31,32]. Selection criteria for these studies were that they provided baseline dietary intake data using consistent methodology as well as body composition. These studies had been approved by the Human Research Ethics Committee at the University of South Australia or CSIRO Human Experimentation Ethics Committee. All volunteers gave written informed consent prior to commencing the trials.

### 2.2. Assessment of Dietary Intake

Dietary intake including total energy from macro and micronutrients was estimated using a 74-item food frequency questionnaire (FFQ) [33] which requests information relating to food choices, frequency, portion size, quantity and consumption rate of different food and beverage items. Participants who were suspected for underestimation or overestimation of daily energy intake (<4000 kJ or >17,000 kJ) were excluded [34]. Detailed information on type and amount and cheese (hard, firm, soft, cottage, ricotta, low-fat), ice-cream, yogurt and reduced fat and full fat milk, including flavoured milk, was collected. The FFQ did not include cream consumption. The FFQ has been validated for use in human dietary intervention trials [35]. The 74 item FFQ was validated against 3 day weighed food records to collect dietary information over one month in *n =* 118 men and women aged between 31 and 74. Mean energy and nutrient intakes were within ±20% difference and classified more than two thirds of the volunteers within ±1 quintile difference for all nutrients [35].

Raw intake scores (total amount in g/day) were provided for each dairy food. The nutrient composition for each product was extracted from the Foodworks Professional nutritional program (Xyris, Qld, Australia) and the energy, macronutrient and micronutrient intake provided from each individual dairy product as a proportion of total daily intake were subsequently determined. Total daily milk intake from all sources was calculated and categorised into full fat or reduced fat. Total dairy product consumption was calculated by summing intakes of all dairy products. 

### 2.3. Anthropometry and Body Composition Assessments

Body composition assessments have been described for each study elsewhere [23,24,25,26,27,28,29,30,31,32]. Briefly, body height was measured to the nearest 0.1 cm with a stadiometer while the participants were barefoot. Body weight was measured to the nearest 0.05 kg with calibrated electronic digital scales while the participants were wearing light clothing and no footwear. Body composition including % body fat and abdominal fat was assessed by using dual-energy X-ray absorptiometry (DXA) (Lunar Prodigy; General Electric, Madison, WI, USA). Body mass index (BMI) was calculated as weight (in kg) divided by height^2^ (in m). Waist and hip circumference were measured according to the International Standards for Anthropometric Assessment to calculate waist/hip ratio (WHR) [36]. Waist to hip ratio was calculated by dividing waist circumference (cm) with hip circumference (cm).

### 2.4. Statistical Analysis

SPSS software (version 17.0; SPSS Inc., Chicago, IL, USA) was used for all statistical analyses. Data were analysed to determine normality of dependent variables by assessing the residual plots of the linear regression analysis. If residual plots were normally distributed then no transformations were performed. If residual plots were not normally distributed they were log transformed and checked for normality. Linear regression was used to explore relationships between total energy intake, and macronutrient intake as well as total dietary calcium and markers of adiposity, with statistical control for age, gender and total energy intake.

Relationships between energy, protein, fat, saturated fat, carbohydrate and calcium (all in g/day) from dairy products with all adiposity measures were also analysed using linear regression. Two models were used: (1) Basic: adjusted for age, gender and total energy intake; and (2) Full: adjusted for Basic covariates + the total dietary intake of each specific macronutrient. For example, when assessing the relationship between dairy calcium and each adiposity measure, total dietary calcium was statistically controlled for.

Absolute intakes of each individual dairy product (full fat and reduced fat milk, total milk, cheese, dairy spreads, yoghurt, and ice cream), as well as total dairy intake, were analysed using the same statistical procedure. Similarly, two models of regression analysis were performed: (1) Basic: adjusted for age, gender, and total energy intake; and (2) Full: adjusted for Basic covariates + other dairy products. For example, when assessing yoghurt intakes, intakes of milk, cheese, ice-cream and dairy spreads were controlled for. The variance inflation factor (VIF) was examined to assess for multicollinearity between variables. As the VIF was <10 for all fully adjusted models, there was no evidence of multicollinearity and subsequently no variables were removed from the analyses.

Additional multivariate analyses were conducted based on the findings from the main analyses, to further explore the relationships between intakes of dairy protein, dairy calcium, and dairy food intake. *p <* 0.05 was considered significant unless otherwise specified.

## 3. Results

### 3.1. Participant Characteristics

After exclusion of 42 participants based on suspected overestimation or underestimation of daily energy intake (<4000 kJ or >17,000 kJ) [34] the final sample totaled 720 participants (367 females and 353 males) aged 51.2 ± 10.4 year, with mean BMI = 32.4 ± 5.7 kg/m^2^ and body fat (by DEXA) = 41.3% ± 8.6% (Table 1). Not all studies carried out the same measurements; hence numbers of participants differ for each measure.

**Table 1:** Characteristics of participants.

	*n*	%
Gender		
Male	353	49
Female	367	51
		*Mean ± SD*
Age (y)	706	51.2 ± 10.4
Height (m)	703	1.70 ± 0.1
Weight (kg)	704	94.2 ± 18.1
BMI (kg/m^2^)	718	32.4 ± 5.7
Waist circumference (cm)	412	105.0 ± 15.8
Hip circumference (cm)	177	117.1 ± 16.0
Waist/hip ratio	177	0.91 ± 0.09
% body fat	347	41.3 ± 8.6
Abdominal body fat (g)	115	4630 ± 1418

### 3.2. Dairy Intakes

Dietary intakes of macro and micronutrients from the total diet and from dairy products are shown in Table 2. The average daily energy intake was 8.4 ± 2.7 MJ. Overall dairy food consumption averaged 386 g/day which equates to approximately one and a half servings of dairy foods per day and accounts for 16% of total energy consumed. Reduced fat milk was the most commonly consumed dairy product (235 ± 200 g/day), followed by whole milk (63 ± 128 g/day), yoghurt (53 ± 66 g/day), cheese (14 ± 13 g/day), ice-cream (13 ± 20 g/day) and spreads (6 ± 11 g/day). 

Supplementary Table S1 presents the mean body composition and dietary intakes of participants across quartile categories of BMI.

**Table 2:** Daily dietary intake of participants.

	Mean ± SD (*n* = 720)
**Energy and macronutrients from total diet and dairy**	
Energy	
Total from diet (MJ)	8.4 ± 2.7
Total from dairy (MJ)	1.3 ± 0.6
% of energy from dairy	16 ± 7
Protein	
Total from diet (g)	103 ± 34
% of total energy	21 ± 3
Total from dairy (g)	19 ± 9
% of total energy from dairy protein	4 ± 2
Fat ^1^	
Total from diet (g)	85 ± 30
% of total energy	37 ± 5
Total from dairy (g)	15 ± 11
% of total energy from dairy fat	7 ± 5
Saturated fat	
Total from diet (g)	33 ± 13
% of total energy	15 ± 3
Total from dairy (g)	9 ± 7
% of total energy from dairy saturated fat	4 ± 3
Monounsaturated fat	
Total from diet (g)	31 ± 11
Total from dairy (g)	4 ± 3
Polyunsaturated fat	
Total from diet (g)	13 ± 6
Total from dairy (g)	0.7 ± 1.2
Carbohydrate	
Total from diet (g)	211 ± 71
% of total energy	40 ± 6
Total from dairy (g)	25 ± 12
**Dairy products and calcium**	
Calcium (mg)	987 ± 326
Total dairy (g)	386 ± 183
Milk (g)	
Whole milk (g)	63 ± 128
Reduced fat milk (g)	235 ± 200
Total Milk (g)	299 ± 170
Cheese (g) ^2^	14 ± 13
Yoghurt (g)	53 ± 66
Ice-cream (g)	13 ± 19
Spreads (g) ^3^	6 ± 11

^1^ Other fats (e.g., trans fats and un-identifiable fatty acids) were not included in the dietary analysis food database; ^2^ Includes hard, firm, soft, cream, ricotta, cottage and low fat cheeses; ^3^ Includes butter and butter blends.

**Table 3:** Associations between total energy, macronutrients, micronutrients and dairy products and BMI, % body fat and abdominal fat.

	Covariate set	Adiposity measure					
Total energy, macro- and micronutrients		BMI (kg/m^2^)		Body fat (%)		Abdominal fat (g)	
		B	95% CI	B	95% CI	B	95% CI
Total energy (MJ/day)	Basic	0.281 **	0.120, 0.442	−0.075	−0.273, 0.124	133 *	31.7, 234
Total protein (g/day)	Basic	0.050 ***	0.024, 0.076	0.017	−0.015, 0.050	−5.28	−22.0, 11.4
Total fat (g/day)	Basic	0.102 ***	0.066, 0.138	0.118 ***	0.075, 0.162	29.5 *	7.05, 52.0
Total CHO (g/day)	Basic	−0.044 ***	−0.057, −0.031	−0.038 ***	−0.054, −0.022	−7.95	−16.3, 0.418
Total saturated fat (g/day)	Basic	0.190 ***	0.129, 0.251	0.171 ***	0.096, 0.246	50.3 *	12.1, 88.6
Total calcium (g/day)	Basic	−2.82 ***	−4.39, −1.24	−4.43 ***	−6.33, −2.53	−1374 **	−2339, −410
**Dairy macronutrients ^a^**							
Energy from dairy (MJ/day)	Basic	0.254	−0.521, 1.03	−0.587	−1.54, 0.367	−103	−590, 384
	Full	NA		NA			
Dairy protein (g/day)	Basic	−0.071 **	−0.122, −0.021	−0.117 ***	−0.178, −0.056	−36.2 *	−67.3, −5.04
	Full	−0.093 ***	−0.144, −0.042	−0.128 ***	−0.190, −0.065	−36.1 *	−68.3, −3.94
Dairy fat (g/day)	Basic	0.065 **	0.027, 0.104	0.034	−0.013, 0.081	16.2	−7.68, 40.1
	Full	0.046 *	0.007, 0.086	0.017	−0.033, 0.066	9.30	−15.4, 34.0
Dairy saturated fat (g/day)	Basic	0.102 **	0.040, 0.165	0.052	−0.025, 0.130	26.3	−13.1, 65.8
	Full	0.018	−0.058, 0.093	0.004	−0.090, 0.099	−4.80	−51.9, 42.3
Dairy CHO (g/day)	Basic	−0.052 **	−0.087, −0.018	−0.073 **	−0.115, −0.031	−17.6	−39.2, 3.95
	Full	−0.037 *	−0.073, −0.001	−0.059 **	−0.102, −0.016	−15.8	−38.3, 6.80
Dairy calcium (g/day)	Basic	−2.19 **	−3.69, −0.690	−3.43 ***	−5.26, −1.61	−1038 *	−1967, −108
	Full	−4.68 *	−8.29, −1.06	−0.156	−4.50, 4.19	−2041	−4340, 257
**Dairy products(g/day) ^b^**							
Total dairy intake	Basic	−0.003 *	−0.005, 0.000	−0.004 *	−0.007, −0.001	−0.821	−2.28, 0.640
	Full	NA		NA		NA	
Full fat milk	Basic	0.003	−0.001, 0.006	0.007 **	0.003, 0.011	1.93	−0.051, 3.92
	Full	0.000	−0.004, 0.003	0.004	0.000, 0.009	1.23	−1.12, 3.58
Reduced fat milk	Basic	−0.003 **	−0.005, −0.001	−0.004 **	−0.006, −0.001	−0.958	−2.25, 0.337
	Full	−0.003 *	−0.005, 0.000	−0.002	−0.005, 0.001	−0.531	−2.05, 0.991
Total milk	Basic	−0.002	−0.005, 0.000	−0.002	−0.005, 0.001	−0.260	−1.82, 1.30
	Full	−0.001	−0.004, 0.001	−0.002	−0.005, 0.001	0.145	−1.39, 1.68
Cheese ^c^	Basic	0.000	−0.034, 0.034	−0.028	−0.070, 0.014	−20.5	−41.4, 0.512
	Full	−0.002	−0.036, 0.032	−0.020	−0.061, 0.021	−17.9	−38.8, 2.96
Dairy spreads ^d^	Basic	0.075 ***	0.037, 0.114	0.026	−0.022, 0.074	13.9	−10.5, 38.3
	Full	0.068 **	0.029, 0.107	0.007	−0.040, 0.054	8.90	−15.0, 32.8
Yoghurt	Basic	−0.005	−0.012, 0.001	−0.016 ***	−0.023, −0.008	−5.18 *	−9.08, −1.29
	Full	−0.044	−0.010, 0.003	−0.014 ***	−0.022, −0.007	−4.34 *	−8.28, −0.408
Ice cream	Basic	−0.012	−.034, 0.009	−0.005	−0.032, 0.022	−1.56	−15.27, 12.15
	Full	−0.011	−0.033, 0.011	−0.004	−0.030, 0.022	−1.38	−14.6, 11.9

BMI, body mass index; CHO, carbohydrate; Basic model adjusted for age and gender, total energy intake; ^a^ Full model: adjusted for age, gender, total intake of relevant macronutrient; ^b^ Full model: adjusted for age, gender, total energy intake, remaining dairy products (g/day); ^c^ Includes hard, firm, soft, cream, ricotta, cottage and low-fat cheeses; ^d^ Includes butter and butter blends; Values are B values (unstandardised regression coefficient); * *p* < 0.05; ** *p* < 0.01; *** *p* < 0.001 (linear regression).

**Table 4:** Associations between total energy, macronutrients, micronutrients and dairy products and waist circumference (WC) and hip circumference (HC).

	Covariate set	Adiposity measure			
Total energy, macro- and micronutrients		WC (cm)		HC (cm)	
		B	95% CI	B	95% CI
Total energy (MJ/day)	Basic	1.00 **	0.425, 1.58	0.774	−0.134, 1.68
Total protein (g/day)	Basic	0.092	−0.002, 0.187	0.094	−0.056,.243
Total fat (g/day)	Basic	0.364 ***	0.237, 0.491	0.470***	0.665
Total CHO (g/day)	Basic	−0.128 ***	−0.175, −0.081	−0.178***	−0.249, −0.107
Total saturated fat (g/day)	Basic	0.597 ***	0.380, 0.814	0.703***	0.367, 1.04
Total calcium (g/day)	Basic	−12.0 ***	−17.6, −6.47	−13.8**	−22.5, −5.08
**Dairy macronutrients ^a^**					
Energy from dairy (MJ/day)	Basic	−0.486	−0.326, 2.29	−0.552	−4.93, 3.83
	Full	NA		NA	
Dairy protein (g/day)	Basic	−0.322 ***	−0.501, −0.143	−0.343 *	−0.624, −0.062
	Full	−0.372 ***	−0.552, −0.191	−0.393 **	−0.677, 0.109
Dairy fat (g/day)	Basic	0.175 *	0.039, 0.311	0.219 *	0.005, 0.433
	Full	0.101	−0.040, 0.242	0.135	−0.085, 0.356
Dairy saturated fat (g/day)	Basic	0.268 *	0.043, 0.492	0.332	−0.021, 0.685
	Full	−0.041	−0.310, 0.227	0.034	−0.388, 0.456
Dairy CHO (g/day)	Basic	−0.206 **	−0.329, −0.084	−0.244 *	−0.436, −0.052
	Full	−0.166 *	−0.294, −0.038	−0.182	−0.382, 0.018
Dairy calcium (g/day)	Basic	−9.64 ***	−15.0, −4.31	−10.3 *	−18.7, −1.95
	Full	−18.8 **	−31.7, −5.85	−12.4	−32.7, 7.96
**Dairy products (g/day) ^b^**					
Total dairy intake	Basic	−0.011 **	−0.019, −0.003	−0.010	−0.023, 0.003
	Full	NA		NA	
Full fat milk	Basic	0.018 **	0.007, 0.029	0.026 **	0.009, 0.044
	Full	0.008	−0.006, 0.021	0.017	−0.004, 0.038
Reduced fat milk	Basic	−0.012 **	−0.020, −0.005	−0.014 *	−0.026, −0.002
	Full	−0.009 *	−0.018, 0.000	−0.007	−0.021, 0.006
Total milk	Basic	−0.008	−0.017, 0.001	−0.005	−0.019, 0.009
	Full	−0.049	−0.013, 0.004	−0.002	−0.016, 0.011
Cheese ^c^	Basic	−0.088	−0.209, 0.033	−0.095	−0.286, 0.096
	Full	−0.082	−0.202, 0.039	−0.083	−0.268, 0.103
Dairy spreads ^d^	Basic	0.180 *	0.042, 0.319	0.215	−0.003, 0.432
	Full	0.136	−0.001, 0.274	0.155	−0.058, 0.367
Yoghurt	Basic	−0.034 **	−0.057, −0.012	−0.043 *	−0.078, −0.007
	Full	−0.028 *	−0.051, −0.006	−0.036 *	−0.071, −0.001
Ice cream	Basic	−0.035	−0.114, 0.043	−0.113	−0.235, 0.009
	Full	−0.031	−0.107, 0.045	−0.105	−0.223, 0.013

BMI, body mass index; CHO, carbohydrate; Basic model adjusted for age and gender, total energy intake; ^a^ Full model: adjusted for age, gender, total intake of relevant macronutrient; ^b^ Full model: adjusted for age, gender, total energy intake, remaining dairy products (g/day); ^c^ Includes hard, firm, soft, cream, ricotta, cottage and low-fat cheeses; ^d^ Includes butter and butter blends; Values are B values (unstandardised regression coefficient); * *p* < 0.05; ** *p* < 0.01; *** *p* < 0.001 (linear regression).

### 3.3. Dietary Intake and Adiposity Measures

#### 3.3.1. Macronutrients and Adiposity

Total energy intake was positively associated with BMI, abdominal fat and WC, when controlling for age and gender (Table 3 and Table 4). With adjustment for age, gender, and total energy intake, total dietary protein intake (g/day) was positively associated with BMI. Total fat and saturated fat intakes from all dietary sources were associated with all adiposity measures (all *p <* 0.05). Carbohydrate intake was inversely associated with BMI, % body fat, WC and HC (all *p <* 0.001). Total dietary calcium was also inversely associated with all measures of adiposity (all *p <* 0.01).

#### 3.3.2. Dairy Macronutrients and Adiposity

Dairy protein (g/day) was inversely associated with all measures of adiposity (all *p <* 0.05), controlling for age, gender, total energy intake, and total protein intake. Dairy-derived calcium was similarly associated with all measures in the basic model; significant associations remained with BMI and WC when adjusted for total calcium intake (both *p <* 0.05). Dairy fat and saturated fat were both positively associated with BMI and WC (*p* < 0.05, basic models). Dairy carbohydrate was inversely associated with BMI, % body fat and WC (*p* < 0.05) with control for total carbohydrate intake.

#### 3.3.3. Dairy Foods and Adiposity

Total dairy food intake (g/day) was inversely associated with BMI, % body fat and WC (all *p <* 0.05), with adjustment for age, gender and total energy intake. Analyses of individual dairy foods showed that consumption of full fat milk was positively associated with % body fat, WC and HC (all *p <* 0.001, basic model only). Reduced fat milk intake was associated with lower BMI, % body fat, WC and HC (all *p <* 0.05). With the added control for intake of other dairy foods, reduced fat milk remained significantly associated with both BMI and WC (both *p <* 0.05). Yoghurt consumption was inversely associated with % body fat, abdominal fat, WC and HC, and these remained with full adjustment for intakes of other dairy products (all *p <* 0.05). Spreads (butter and butter blends) were positively associated with BMI, and WC (basic model, *p <* 0.05).There was no relationship between cheese or ice-cream consumption and adiposity.

#### 3.3.4. Additional Dairy and Adiposity Analyses

To further explore the relationships between intakes of dairy protein, dairy calcium, and dairy food intake, additional multivariate analyses were conducted. Dairy protein remained significantly and inversely associated with % body fat (*p* < 0.001), abdominal fat (*p* = 0.001), WC (*p* = 0.003), and HC (*p* = 0.007), with control for age, gender, total energy intake, and total dairy food intake. Dairy calcium remained significantly and inversely associated with BMI (*p* < 0.05), % body fat (*p* < 0.001), abdominal fat (*p* < 0.001), WC (*p* = 0.001), and HC (*p* = 0.003), with control for age, gender, total energy intake, and total dairy food intake (data not shown). 

Yoghurt intake and total dairy food intake remained inversely associated with % body fat (both *p <* 0.05), with addition of dairy protein and dairy calcium to the model. Reduced fat milk was no longer significantly associated with BMI or WC with the addition of dairy protein and dairy calcium (data not shown).

## 4. Discussion

In this cross-sectional study of overweight/obese Australian adults, dairy protein, dairy calcium, total dairy food intake, and a number of individual dairy foods (namely yoghurt and reduced fat milk) were inversely associated with a number of adiposity measures. Relationships between dairy protein and % body fat, abdominal fat, and WC remained with full adjustment for total energy intake and total dairy food intake. Yoghurt intake and total dairy food intake remained inversely associated with % body fat (both *p <* 0.05), with addition of dairy protein and dairy calcium to the model. These findings are consistent with our hypothesis that higher intakes of dairy foods, as well as dairy protein and calcium would be associated with lower levels of adiposity. 

Our study sample were consuming more dairy products than in Australia’s National Nutrition Survey [1], in which men and women aged 19 years and over consumed 322 g and 258 g of milk products and other dairy foods per day, respectively. Interestingly, the average consumption in our population was still less than the recommended dietary intake (RDI) for dairy products (2–3 servings per day; serving sizes milk 250 mL, yoghurt 200 g, cheese 40 g, custard 250 mL) in Australia [37]. 

The novel aspect of our study is that we explored relationships between intakes of specific dairy food components and direct markers of adiposity as measured by DEXA. Other studies have used indirect markers of adiposity such as BMI and have not necessarily focused on consumption of dairy foods but on nutrients such as calcium. Data from the present study is in agreement with a number of other observational and dietary intervention studies which have shown beneficial effects of dairy product consumption on body composition or inverse associations with dairy product consumption and body composition [8,11]. For example, in the Coronary Artery Risk Development in Young Adults (CARDIA) study a higher dairy food intake (consumed dairy foods ≥35 times per week) was associated with lower levels of obesity (BMI) in individuals who were overweight at baseline compared with lower daily intakes of dairy products. This relationship did not exist in individuals who had a BMI of <25 kg/m^2^ at baseline [38]. In the present study we showed dairy protein, dairy calcium, and reduced fat milk were associated with lower BMI. Moreover, in a weight loss/weight maintenance study by Champagne *et al*. [39], increasing low fat dairy food intake was associated with significant weight loss during the weight maintenance phase (−0.17 kg per 6 months per 1-serving increase). However, Chen and colleagues [40] conducted a meta-analysis of the effects of dairy food intake on body weight and body fat in 29 studies with *n =* 2101 participants and showed that dairy food consumption did not lead to a significant reduction in weight. However, where diets were energy restricted, or in studies of less than 1 year duration, dairy food consumption reduced body weight. Studies that were of *ad libitum* design or of greater than 1 year duration did not result in reduced body weight following dairy food consumption. Interestingly our data showed total dairy intake, yoghurt, and dairy protein were inversely associated with % body fat, and yoghurt and dairy protein were inversely associated with abdominal fat. Furthermore a study by Rajpathak *et al*. [9,41] in a 12 year follow up from the US Health Professional’s Study examining relationships between dairy food and calcium intake and weight status showed in men who increased their dairy food intake the most, compared with men who decreased their dairy food intake the most, a small increase in weight (3.1 compared with 2.6 kg; p for trend = 0.001).The authors state that the association was primarily due to an increase in high-fat dairy food intake as low-fat dairy food consumption was not significantly associated with weight change. Interestingly the authors found no association with calcium intake and weight change, which does not support current evidence that calcium from dairy food is thought to be the responsible component for the benefits with reduced adiposity however this is controversial. 

In the present study we showed negative associations between calcium intake and measures of adiposity which further supports previous research showing benefits of calcium supplementation on body weight [41]. Previous research has shown that calcium may increase faecal excretion of fatty acids, including saturated fat and bile acids, minimising effects on serum cholesterol and increasing energy loss which may impact on measures of adiposity [42,43] and also influence energy partitioning through lipogenesis and lipolysis. In a study by Zemel and colleagues [44] where participants were randomised to either a low dairy food diet (<1 serving dairy products/day) or a recommended dairy food diet (>3 servings dairy products/day) subsequent to weight loss, those on the latter diet exhibited evidence of greater fat oxidation and were able to consume more energy without greater weight gain than the low dairy group. Similarly, in another study by Zemel *et al*. [45] demonstrated a 9% loss of body weight following a high calcium diet and an 11% loss of body weight on a high dairy food diet following a 6 month energy restricted diet. Despite the energy restriction, there was a greater fat loss from the trunk region on the high dairy food calcium diet, supporting the role of intracellular calcium and energy partitioning. In contrast a study by Bowen *et al*. [46] showed no difference in the amount of weight lost in an energy restricted high dairy protein diet (high calcium 2400 mg/day) *vs*. high meat protein (low calcium 500 mg/day) diet.

Besides calcium, dairy products contain a range of nutrients including proteins (whey and casein), branched chain amino acids and peptides. Data from our study showed an inverse relationship with dairy protein and all adiposity measures, with control for total dietary protein. These data support previous research which has indicated that dairy protein might be the component responsible for beneficial effects on body composition [16,47]. These potential effects may be related to influence on adipocyte lipid metabolism or more specifically due to increased diet induced thermogenesis and subsequently greater energy expenditure and less fat storage. Another potential mechanism related to dairy consumption and improved body composition may be around the current evidence supporting dairy protein consumption, and the role of dairy in satiety relating to weight loss and weight gain prevention [48,49]. Dairy foods are predominantly casein (80%) while whey makes up 20% which have different gastric emptying rates which subsequently impacts satiety. There in fact may be other effects of dairy protein on satiety hormones such as cholecystokinin and peptide YY and hunger stimulating hormone ghrelin however the evidence is less clear. These hormones were not measured in the present study. While growing evidence of the potential beneficial effects of dairy food consumption and obesity remains controversial, data from the current study suggest the consumption of dairy products and more specifically dairy protein and calcium derived from low fat dairy foods, such as milk and yoghurt, are beneficially associated with indices of body composition in an overweight/obese population. These findings support contemporary Australian public health dietary recommendations [37,50]. This is the first study that has examined this question within such a sample as prior studies have examined a wider cross section inclusive of individuals of normal BMI. Nevertheless, given that the present study was cross-sectional, we cannot allude to cause/effect and indeed clinical interventions have shown variable results in terms of *ad libitum* or energy restricted diets, duration and fat content of dairy foods. In presenting these data we recognise numerous limitations associated with not only cross-sectional studies, but the available published evidence relating to the quality of collected dietary information and choice of dietary tools, use of accurate biomarkers of dairy fat intake, nutrient composition of bovine milk, statistical adjustment of potential confounders and the fact that dairy foods are consumed as part of a diet and not in isolation. Given the complexity of studying nutrition, dairy food intake and obesity it is difficult to draw conclusions without conducting long term dietary intervention trials without energy restriction to investigate whether dairy foods exert beneficial effects on body composition. We also acknowledge the difficulty in drawing data together from different studies and two clinical trial centres and its representativeness of the general population.

## 5. Conclusions and Implications

These data provide additional evidence that dairy foods may not unfavourably influence body composition in an overweight/obese population. Albeit this was a cross-sectional study utilising data from two clinical trial centres, we recognise the limitations associated with the nature of this study, however these results, together with data from intervention studies and epidemiological studies, should be considered when evaluating the evidence in preparation of evidence statements and dietary guidelines. Future studies should focus on collected detailed information on a variety of dairy foods to include total amount as well as fat content, in addition to collecting information on markers of adiposity and potential confounding factors.

## Supplementary Information

**Table S1:** Body composition and dietary data across quartile categories of BMI.

Variable	Quartile categories of BMI ^a^								*P* ^b^
	1 *n* = 179		2 *n* = 180		3 *n* = 180		4 *n* = 179		
	M	SD	M	SD	M	SD	M	SD	
Age	53.4	12.0	51.7	8.5	51.7	9.7	48.2	10.8	<0.001
Gender (*n* %)									0.047
Male	87	24.7	93	26.4	99	28.1	73	20.7	
Female	92	25.1	87	23.8	81	22.1	106	29.0	
**Body composition variables**									
Body weight (kg)	75.8	11.0	89.1	10.4	97.5	11.7	113	14.8	<0.001
% body fat	34.8	6.2	38.4	8.2	39.6	7.3	46.5	7.6	<0.001
Abdominal fat (g)	2781	662	3684	988	4370	681	5537	1218	<0.001
Waist Circumference (cm)	87.6	11.2	101	7.8	109	8.6	121	10.7	<0.001
Hip Circumference (cm)	100	6.3	112	5.4	115	5.4	133	12.9	<0.001
Waist/hip ratio	0.85	0.08	0.91	0.06	0.96	0.09	0.93	0.08	<0.001
**Dietary variables**									
Total energy (MJ/day)	7.8	2.5	8.5	2.7	8.5	2.7	8.8	2.6	0.006
Total protein (g/day)	95.5	31.1	103	32.9	105	34.0	111	36.8	<0.001
Total fat (g/day)	75.4	29.2	84.4	30.6	87.0	30.5	91.7	29.8	<0.001
Total CHO (g/day)	207	68.6	214	74.7	210	71.8	212	70.7	0.83
Total saturated fat (g/day)	28.6	12.2	33.3	13.4	34.9	13.4	36.9	12.6	<0.001
Total calcium (mg/day)	988	317	1021	353	962	297	980	337	0.37
**Dairy macronutrients**									
Energy from dairy (MJ/day)	1.2	0.6	1.3	0.6	1.3	0.6	1.3	0.6	0.17
Dairy protein (g/day)	19.0	8.6	19.7	9.1	18.0	7.9	18.0	8.5	0.19
Dairy fat (g/day)	11.9	9.1	14.8	11.8	15.8	11.5	16.7	11.6	<0.001
Dairy saturated fat (g/day)	7.4	5.7	9.0	7.1	9.7	7.0	10.2	7.0	0.001
Dairy CHO (g/day)	25.7	12.5	25.7	12.6	23.4	12.2	23.4	12.1	0.10
Dairy calcium (g/day)	632	287	651	308	592	281	596	281	0.15
**Dairy products (g/day)**									
Total dairy intake	395	182	405	197	367	174	375	177	0.18
Full fat milk	52.1	120	68.1	141	61.0	122	70.8	128	0.52
Reduced fat milk	253	187	249	225	218	191	221	192	0.22
Total milk	305	158	317	195	279	162	292	162	0.18
Cheese ^c^	12.4	9.7	14.8	12.7	15.6	14.8	14.2	12.3	0.09
Dairy spreads ^d^	3.8	7.9	5.7	9.6	7.3	11.7	8.3	12.2	<0.001
Yoghurt	59.7	67.1	54.4	67.2	51.0	68.3	47.3	62.3	0.34
Ice cream	13.7	20.5	13.6	19.4	14.1	21.2	12.6	16.3	0.90

^a^ Quartile categories of BMI were as follows: 1: <28.5 kg/m^2^; 2: 28.5–31.8 kg/m^2^; 3: 31.9–35.9 kg/m^2^; 4: ≥36.0 kg/m^2^; ^b^ *P* for between group differences according to analysis of variance; ^c^ Includes hard, firm, soft, cream, ricotta, cottage and low-fat cheeses; ^d^ Includes butter and butter blends.
